# A comparative study between the imaging system and the optical tracking system in proton therapy at CNAO

**DOI:** 10.1093/jrr/rrt043

**Published:** 2013-07

**Authors:** Maxime Desplanques, Barbara Tagaste, Giulia Fontana, Andrea Pella, Marco Riboldi, Giovanni Fattori, Andrea Donno, Guido Baroni, Roberto Orecchia

**Affiliations:** 1 Bioengineering Unit, Clinical Department, Fondazione Centro Nazionale di Adroterapia Oncologica (CNAO), Strada Campeggi, 53 - 27100 Pavia, Italy; 2 PARTNER Project Researcher, CERN, Route de Meyrin, 385 - 1217 Meyrin Switzerland/France); 3 Department of Electronics, Information and Bioengineering, CartCasLab, Politecnico di Milano, Via G. Colombo, 40 - 20133 Milan, Italy; 4 Department of Bioengineering, Università di Pavia, Via Adolfo Ferrata, 1 - 27100 Pavia, Italy; 5 Istituto Europeo di Oncologia, Via Ripamonti, 435 Milano - P.I. 08691440153, Italy – Fondazione CNAO, Strada Campeggi, 53 - 27100 Pavia, Italy

**Keywords:** IGRT, patient positioning, optical tracking system, particle therapy, head and neck, pelvis localizations

## Abstract

The synergy between in-room imaging and optical tracking, in co-operation with highly accurate robotic patient handling represents a concept for patient-set-up which has been implemented at CNAO (Centro Nazionale di Adroterapia Oncologica). In-room imaging is based on a double oblique X-ray projection system; optical tracking consists of the detection of the position of spherical markers placed directly on the patient's skin or on the immobilization devices. These markers are used as external fiducials during patient positioning and dose delivery. This study reports the results of a comparative analysis between in-room imaging and optical tracking data for patient positioning within the framework of high-precision particle therapy. Differences between the optical tracking system (OTS) and the imaging system (IS) were on average within the expected localization accuracy. On the first 633 fractions for head and neck (H&N) set-up procedures, the corrections applied by the IS, after patient positioning using the OTS only, were for the mostly sub-millimetric regarding the translations (0.4±1.1 mm) and sub-gradual regarding the rotations (0.0°±0.8°). On the first 236 fractions for pelvis localizations the amplitude of the corrections applied by the IS after preliminary optical set-up correction were moderately higher and more dispersed (translations: 1.3±2.9 mm, rotations 0.1±0.9°). Although the indication of the OTS cannot replace information provided by in-room imaging devices and 2D-3D image registration, the reported data show that OTS preliminary correction might greatly support image-based patient set-up refinement and also provide a secondary, independent verification system for patient positioning.

## INTRODUCTION

Over recent years, the huge increase in intensity-modulated radiation therapy (IMRT), arc therapy and tomotherapy clinical treatments has demanded higher accuracy in patient positioning, especially within the framework of therapy design envisaging dose hypofractionation or dose escalation [1]. These technical advancements, known under the general term IGRT (imaging-guided radiotherapy), like low-energy imaging (kV) or CBCT (cone beam computed tomography), have been shown to improve the quality of the daily radiotherapy workflow. All of this progress has focused clinical applied research on intrafractional tumor tracking [2–4], with ‘gating’ or ‘tracking’ techniques being investigated and clinically applied in order to increase the accuracy of moving lesions treatment.

X-ray imaging and infrared optical tracking provide information of a different nature. The imaging system (IS) provides data describing patient bony structures visualized on radiological images obtained from a coupled X-ray tube and radiation detector. (Soft tissue volumetric visualization is obtained from CBCT [5].) These images are fed to image registration software for the quantification of the mismatch between current and reference images and the elaboration of a position correction vector for patient-set-up improvement that gives the positioning error based on the internal structures of the patient. Conversely, infrared optical tracking (i.e. optical tracking system, OTS) features real-time detection of the 3-D position of surface fiducials fixed on the patient body surface (directly on the skin, or on the immobilization devices). Robust stereotactic algorithms [6] or external/internal motion correlation models [7–8] are applied to estimate the position of the target from the real-time data flow of the 3-D surrogate position. Although optical tracking finds its most usual application for respiratory motion management in time-resolved radiation therapy, the intrinsic accuracy in 3-D localization of surface fiducials is particularly valuable for precise patient-set-up through appropriate integration with in-room imaging technologies [9] and continuous immobility verification.

In this study, we assessed the performance of an integrated optical- and image-based system for patient set-up, as routinely used at the Centro Nazionale di Adroterapia Oncologica (CNAO) for cranial and extra-cranial irradiation of active scanned proton and ion beams. The aim was to compare the data describing patient set-up correction provided by the two system components with a view to optimizing the combined use of optical tracking and X-ray imaging for reducing dose to patient and set-up time.

## MATERIALS AND METHODS

**Fig. 1.**
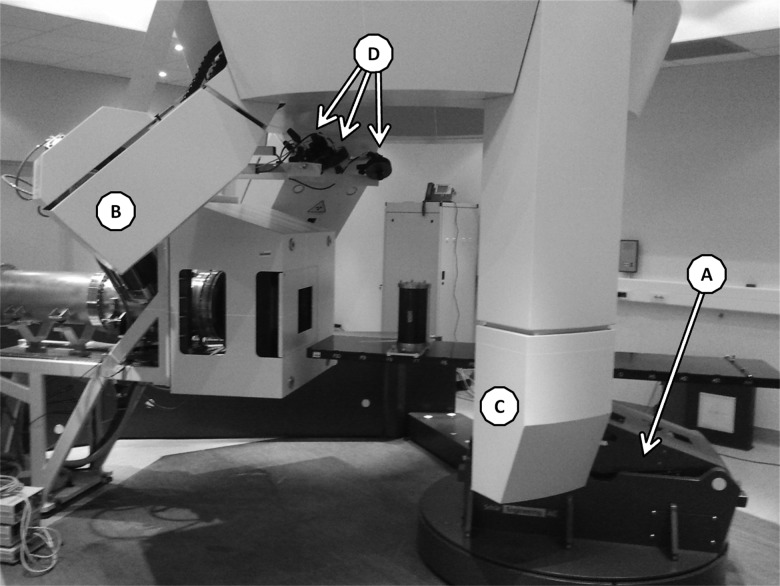
One of the three treatment rooms at CNAO. Each room is composed of three different devices. The Patient Positioning System (PPS) (**A**) on which is docked the couch, the Patient Verification System (PVS), an imaging system fixed to the ceiling from which kV X-ray tubes (**B**) and flat panels (**C**) are deployed, and the Optical Tracking System (OTS) (**D**) composed of three infrared cameras mounted on the nozzle and detecting the positions of reflecting markers fixed on the patient's contention device.

Figure 1 depicts the components of the integrated patient positioning systems installed in CNAO treatment rooms. The single devices are technically described below.

### Patient positioning system

In order to drive the patient with high accuracy in the nominal treatment position, CNAO treatment rooms are equipped with robotic patient positioning systems (PPSs), which feature highly accurate six-degrees-of-freedom patient handling in a large working volume. The systems were manufactured by Schaër Engineering AG (Flaach, Switzerland). The design of the PPS is based on pantographic architecture, thus avoiding the drawbacks of kinematics serial manipulators in featuring high positional open-loop accuracy with varying weight. The PPS translates over a granite basement with 0.1-mm evenness, according to manufacturer's certification. The system features the automatic docking of either a treatment couch or chair from a manual transport system, thus allowing patient preliminary set-up outside the therapy bunker. The user interface for PPS steering is installed both inside the treatment room and in the local control room. Ranges of motion of PPS movements are as follows (with respect to the room isocenter): latero-lateral and longitudinal translations: *x* ± 1000 mm, *y* ± 1000 mm related to the isocenter of the room, respectively; vertical translation: *z* = 600 mm (from 700–1300 mm above ground); pitch: ±30°, roll: ±15°, and yaw: ± 120°. Motion accuracy was assessed to fulfill the manufacturer's specifications (0.3 mm peak linear error) within the working volume (±1000 mm around the isocenter), during repeated measurement campaigns performed by an independent company and by the CNAO staff. In both cases, a Leica Laser Tracker (angle resolution: 0.14", distance resolution: 1.26 µm) was used to quantify the PPS motion accuracy [10].

### Patient verification system

Two of the three treatment rooms at CNAO are equipped with a rotating stereoscopic X-ray imaging system, called a patient verification system (PVS). When imaging is needed during patient set-up, two orthogonal low-kV X-ray tubes and related high-resolution flat panels are deployed from the main structure to reach predefined imaging positions, and two contemporary images are captured.

The deployment repeatability of the PVS components was assessed to be 0.15 mm, and the peak uncertainties in the rotation of the whole structure were found to be < 0.1°. These measurements were performed by means of the same measurement device that was used to check the PPS accuracy (Leica Laser Tracker) [10] and fulfilled the specifications provided by the manufacturer.

PVS imaging components consist of a double couple of X-ray tubes and flat panels. The X-ray tube (Varian A277) allows acquisition of images from 40–150 kVp and from 0.1–500 mAs. The two digital flat panel receptors (Varian PaxScan 4030E) feature a 3200 × 2304 pixel resolution for a sensible area of 40 × 30 cm.

The calculation of the patient set-up correction vector was obtained by a 2D-3D rigid image registration procedure implemented in the certified commercial application Verisuite (MedCom GmbH, Darmstadt, Germany). X-ray images captured during patient set-up were matched automatically (after manual preliminary alignment) to the corresponding digitally reconstructed radiographs (DRRs) generated from the treatment planning CT (TPCT), according to the known (and calibrated) PVS imaging geometry. The iterative image-matching procedure produced a six-degrees-of-freedom roto-translation vector expressed with respect to the room isocentric reference system, that best matched the couple of corresponding images. Once applied to the couch, this corrective motion minimizes the detected misalignments of the bony structures between current patient set-up and reference TPCT.

### Optical tracking system

The optical tracking systems installed at CNAO are equipped with three digital, infrared cameras (∼680 nm), which are fixed on the beam nozzle. Each system features the 3-D localization of passive, reflective markers through real-time processing and triangulation of the image data coming from the cameras. The system working volume measures 500 × 500 × 300 mm^3^ and is centered at the room isocenter. The 70 Hz 3-D dataflow was used for real-time patient position and immobility verification with direct visualization of the current marker displacements to the operators during each phase of patient set-up and treatment delivery. The OTS optical calibration was performed according to a so-called ‘magic-wand’ procedure, based on the epipolar geometry of the stereometric pairs defined by the system TV cameras. The mapping of the OTS reference system (with respect to which the 3-D co-ordinates of the markers are reconstructed) and the isocentric room reference frame were obtained by acquiring a configuration of nine markers placed on a control object, previously measured by a laser tracking device (angle resolution: 0.14", distance resolution: 1.26 µm). The same object is used for daily Quality Assurance (QA) of the OTS and PVS systems, as it carries optical and radio-opaque references visible on both systems. QA procedure envisages the placing of the control object on the PPS in a predefined configuration, such that the center of the object coincides with the room isocenter, followed by a PVS and OTS acquisition of the respective references and comparison with respect to corresponding reference position. OTS markers deviations < 0.5 mm are usually experienced in daily QA. In case deviations greater than 0.7 mm are detected, a new OTS calibration is performed (for which 30 min is required). At CNAO, the OTS is used for quantitative patient set-up verification and correction, and for patient immobility verification, and compares the real-time acquired marker positions with the corresponding reference positions extracted from the CT scans used for treatment planning [11]. During patient set-up, an automatic point-based registration procedure is applied for the real-time estimation of the corrective six-degrees-of-freedom roto-translation vector, which minimizes current marker displacements with respect to reference. At user discretion, the vector can be sent to the PPS steering system automatically for correction implementation. Although the OTS could be efficaciously applied for continued detection of respiratory motion, this capability has not yet been exploited clinically for pelvic treatments, the CT scanning to date being performed in free-breathing conditions.

### Protocols and patients selected for this study

The CNAO is a new particle therapy center, and to date only a few medical protocols have been validated by the medical ethical committee. The pathologies treated in Head and Neck (H&N) have been squamous cell carcinomas, chondrosarcomas and chordomas. Our database is composed of 633 fractions, coming from 21 patients. All of them received a fractionation of 2 GyE/fraction, to a total dose of from 10 GyE (re-irradiation) to 74 GyE. The only pathology treated in the pelvic area has been chordoma, with a fractionation of 2 GyE/fraction. This sample is composed of 236 fractions coming from eight patients, for whom a total dose of 74 GyE was prescribed.

### Preparation of the treatment

**Fig. 2.**
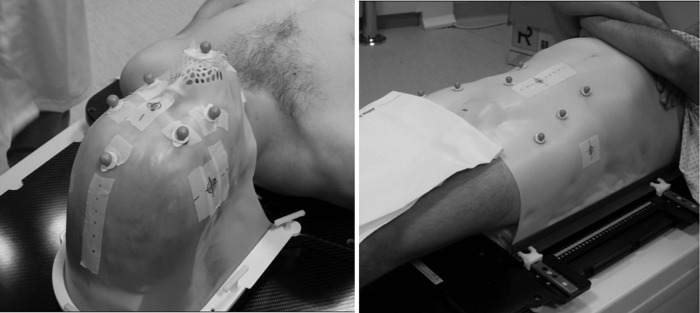
The patient to be simulated or treated is immobilized by the immobilization device (a mask for head and neck regions, on the left of the image – a cast for pelvic regions, on the right of the image) fixed to the couch. The markers (six for head and neck regions, seven for pelvic regions) are fixed on the mask/cast in a position that will be visible to the three infrared cameras in the treatment room, in order to be exploitable by the OTS.

The treatment simulation at CNAO takes place at the CT unit and consists of four main phases: immobilization cast manufacturing, radio-opaque surface markers fixation on the cast, patient CT scanning, and virtual simulation. In the first step, the mask (or cast) is bathed in hot water until it reaches a temperature that makes it malleable enough to fit around the patient shape and to be fixed to the couch. Roughly 10 min are required to let the mask cool down and become rigid, memorizing the patient shape. Once the mask is rigid, radio-opaque markers (visible on the CT slices) are fixed onto it, taking care to give rise to a marker configuration visible to the three OTS infrared cameras installed in the treatment rooms. Usually, six markers are fixed for H&N treatments, and seven markers for pelvic treatments (two exemplifying clinical cases are shown in Fig. 2).

Finally, the region to be treated is defined by the radiation oncologist and marks are drawn on the mask/cast (for pelvic treatments, the patient is also tattooed), in correspondence with laser lines intersection with the patients and the immobilization device. Once CT scanning has been completed, the center of the volume to be treated is determined from CT geometry, and new marks are drawn on the cast to represent the projection of the isocenter on the mask/cast (virtual simulation), serving as a reference for patient preliminary alignment on the treatment couch during treatment fraction preparation.

### Patient positioning workflow

At CNAO, patient preliminary positioning takes place in a dedicated room, in order to spare bunker time and optimize patient throughput. In the preparation room, the patient lies down on the couch and is manually aligned using in-room lasers and tattooed references. After visual alignment, the contention device is put into operation, and the patient is brought into the treatment room by means of a manual transport system compatible with the PPS docking mechanism. After the docking, the PPS moves to the nominal set-up configuration.

**Fig. 3.**
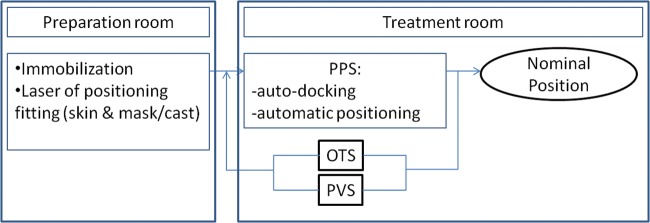
Patient workflow performed at the CNAO before the dose delivery. The patient is immobilized outside the treatment room and afterwards is introduced into the treatment room, docked to the PPS and driven to the planned position for treatment. Only then, corrections (firstly from the OTS and secondly by the PVS) are applied and repeated if needed. Once this process is concluded, the patient is in the expected (or nominal) position and the dose can be delivered.

The set-up verification and correction procedure is split up into two phases (Fig. 3): first, the real-time estimated corrective roto-translation provided by the OTS is sent to the PPS for surface markers displacement minimization; second, the X-ray imaging device (PVS) is put into operation and a new correction vector based on bony anatomy mismatch minimization is calculated for patient set-up refinement. This refining correction is applied only if size of corrective translation and rotations are higher than 0.5 mm and 0.5°, respectively. If needed, the image-based correction is repeated at the clinician's discretion until a satisfactory geometrical set-up is obtained. After the above-described double set-up verification and correction procedure, beam delivery is commenced and the OTS is used for continuous patient set-up monitoring for all treatment fields. At CNAO, the patient positioning workflow is identical for H&N and pelvis localizations.

## RESULTS

Tables 1 & 2 report the size of corrections and respective residuals after the application of the double corrective actions during the patient set-up.

**Table 1.** Size of set-up corrections and respective residuals proposed by OTS and PVS

	OTS correction	OTS residuals	Imaging correction	Imaging residuals	OTS discrepancy
	<Δ> ± σ	<Δ> ± σ	<Δ> ± σ	<Δ> ± σ	<Δ> ± σ
	[min/max]	[min/max]	[min/max]	[min/max]	[min/max]
CC (mm)	–0.31 ± 1.40	–0.01 ± 0.25	0.66 ± 1.11	0.15 ± 0.47	–0.60 ± 1.14
	[–4.4/ + 4.3]	[–1.1/ + 2.5]	[–3.60/ + 5.31]	[–2.00/ + 2.40]	[–4.9/ + 2.6]
LL (mm)	0.65 ± 1.41	0.05 ± 0.35	0.76 ± 0.79	0.10 ± 0.45	–0.68 ± 0.67
	[–4.0/ + 4.5]	[–1.8/ + 1.9]	[–2.24/ + 3.79]	[–1.66/ + 2.03]	[–4.5/ + 2.6]
AP (mm)	–0.63 ± 1.00	0.00 ± 0.13	–0.37 ± 1.08	–0.03 ± 0.29	0.50 ± 1.14
	[–3.0/ + 2.6]	[–1.5/ + 0.7]	[–4.24/ + 3.93]	[–1.30/ + 1.70]	[–3.2/ + 4.1]
Pitch (°)	0.20 ± 0.34	0.00 ± 0.14	0.25 ± 0.80	–0.06 ± 0.45	–0.10 ± 0.84
	[–1.9/ + 1.4]	[–1.1/ + 0.8]	[–3.16/ + 3.00]	[–2.26/ + 2.08]	[–3.0/ + 2.5]
Roll (°)	0.26 ± 0.45	0.00 ± 0.11	–0.42 ± 0.84	0.00 ± 0.33	0.48 ± 0.73
	[–1.6/ + 1.4]	[–0.3/ + 1.3]	[–3.97/ + 1.92]	[–2.08/ + 1.14]	[–1.2/ + 3.9]
Rotate (°)	–0.01 ± 0.33	0.00 ± 0.10	0.03 ± 0.71	0.09 ± 0.33	–0.17 ± 0.72
	[–0.9/ + 1.6]	[–1.2/ + 0.5]	[–2.90/ + 2.87]	[–1.21/ + 1.46]	[–3.0/ + 3.1]

Data coming from the first 633 treatment sessions in H&N localization are reported. CC = Cranio-caudal, LL = Latero-lateral, AP = Antero-posterior.

The amplitude of OTS corrections was (mean ± standard deviation) –0.1 ± 1.4 mm and 0.1 ± 2.7 mm in H&N and pelvic tumor treatments, respectively; corresponding rotational correction sizes were 0.1 ± 0.4° (for H&N), and 0.0 ± 0.4° (for pelvis). The residual discrepancies after correction application by moving the PPS according to the suggested correction vector were mostly negligible (<0.2 mm, <0.1°) (second column in Table 1 and Table 2). Image-based 2D-3D registration, applied after the preliminary optically guided set-up correction, produced correction vectors of average size in H&N patients, measuring 0.4 ± 1.1 mm and 0.0 ± 0.8° for the translational and rotational components, respectively. The corresponding translational and rotational image-based corrections were 1.3 ± 2.9 mm and 0.1 ± 0.9° for pelvis localizations (third column in Tables 1 and 2). The residuals after correction application were of negligible size (fourth column in Tables 1 and 2). Finally, the discrepancy between the OTS and the PVS corrective indications (quantified as the set-up correction suggested by the OTS at the end of the image-based set-up refinement) is reported in the last column (OTS discrepancy) of Tables 1 and 2. This value is put forward to represent the margin of uncertainty associated with OTS indications with respect to the image-based system (considered as the reference). This information is made available to clinicians, in order to reduce to a bare minimum the recourse to imaging during patient set-up.

**Table 2.** Size of set-up corrections and respective residuals proposed by OTS and PVS

	OTS correction	OTS residuals	Imaging correction	Imaging residuals	OTS discrepancy
	<Δ> ± σ	<Δ> ± σ	<Δ> ± σ	<Δ> ± σ	<Δ> ± σ
	[min/max]	[min/max]	[min/max]	[min/max]	[min/max]
CC (mm)	0.40 ± 1.29	–0.03 ± 0.40	0.22 ± 3.70	0.05 ± 1.14	–0.17 ± 3.65
	[–2.6/ + 4.0]	[–1.8/ + 3.1]	[–8.21/ + 9.49]	[–2.37/ + 3.96]	[–9.3/ + 8.1]
LL (mm)	–1.42 ± 3.61	0.09 ± 0.77	1.92 ± 2.57	–0.06 ± 1.41	–1.39 ± 2.52
	[–11.4/ + 10]	[–3.9/ + 1.9]	[–4.65/ + 9.60]	[–3.79/ + 4.16]	[–7.7/ + 4.93]
AP (mm)	1.44 ± 1.65	0.28 ± 0.69	1.72 ± 1.89	–0.17 ± 0.84	–1.07 ± 1.80
	[–3.1/ + 5.0]	[–1.9/ + 3.2]	[–3.28/ + 9.89]	[–3.35/ + 3.60]	[–6.7/ + 3.8]
Pitch (°)	–0.04 ± 0.56	–0.03 ± 0.11	0.12 ± 0.91	0.00 ± 0.64	–0.06 ± 0.85
	[–1.8/ + 1.8]	[–0.5/ + 0.3]	[–5.80/ + 2.35]	[–1.77/ + 2.04]	[–3.0/ + 2.2]
Roll (°)	–0.01 ± 0.39	0.01 ± 0.14	0.18 ± 0.99	0.03 ± 0.44	–0.15 ± 0.81
	[–1.2/ + 1.3]	[–0.5/ + 0.5]	[–2.49/ + 4.92]	[–1.24/ + 1.78]	[–2.4/ + 1.9]
Rotate (°)	–0.07 ± 0.20	0.00 ± 0.08	0.04 ± 0.66	–0.04 ± 0.56	–0.10 ± 0.64
	[–1.1/ + 0.5]	[–0.6/ + 0.2]	[–2.22/ + 2.37]	[–2.08/ + 1.12]	[–3.0/ + 1.6]

Data coming from the first 236 treatment sessions in pelvis localization are reported. CC = Cranio-caudal, LL = Latero-lateral, AP = Antero-posterior.

## DISCUSSION

In this work, we reported the results of a comparative analysis focused on the indications provided by two different devices installed in the treatment rooms at CNAO for patient set-up verification and correction. The six-degrees-of-freedom correction vectors provided by the OTS were estimated by means of a 3-D real-time comparison between the current position of surface fiducials (fixed on patient immobilization masks) and the corresponding reference obtained from TPCT. Despite the high intrinsic accuracy of optical devices and marker segmentation procedures in CT slices [11], the information is affected by large uncertainties due to relative motion of patient anatomy with respect to the immobilization device, on which the surface markers are fixed. Therefore, OTS-driven correction is considered to be a preliminary patient set-up correction and a rapid check of the absence of significant anomalies in current treatment geometry within the framework of the OTS usage, i.e. a secondary, independent Record and Verify (R&V) system. At CNAO, image-based set-up verification is the ultimate means for clinical assessment of patient set-up quality before treatment delivery.

The quantification of the residual OTS corrective indications after image-based set-up refinement (at clinical decision to start the treatment) allows one to assess the level of agreement between the two systems. Interestingly enough, despite the above-mentioned limitation of point-based optical tracking, average discrepancies were found to be lower than 1 mm and 2 mm for H&N and pelvic tumor localizations, thus compatible with CTV-PTV margins applied in most of the cases. Conversely, the variability around average values revealed geometrical uncertainties exceeding CTV-PTC margins (2 mm for H&N, 4 mm for pelvis), thus calling for the highest care in balancing OTS and image-based data for patient set-up quality improvement.

The variability of OTS vs image-based indications is mainly influenced by the uncertainties in the repeatability of the contention configuration and positioning; due to the fact that surface fiducials are placed on the immobilization devices, the geometrical repeatability of the contentions device influences OTS measurements and hinders reliance on OTS only for a precise patient set-up (with sub-millimetric accuracy), as required in particle therapy. However, one should also consider the intrinsic accuracy of the image-based registration procedure, which is influenced by the properties and quality of in-room acquired images (field of view, pixel resolution, quantization levels), by the specific region of interest defined by the radiation oncologist for image-based registration, and by the robustness of the 2D-3D image registration algorithm.

In accordance with our expectations, the reported data revealed that the magnitude of the corrections applied for the H&N localizations were smaller by far than the ones required in pelvis localizations. This is explained by the fact that the geometric repeatability of the H&N immobilization device is higher with respect to pelvic casts, and by the influence of patient breathing motion on cast morphology stability, especially in patients treated in the prone position.

As a whole, the reported results account for a residual geometric margin of uncertainties, which is compatible with the CTV-PTV safety margins applied during a treatment plan, although one should be fully aware that bony anatomy may not represent a reliable surrogate for target position and may not account for daily variations of beam path caused by patient anatomo-pathological modifications. In the framework of the CNAO workflow for patient irradiation preparation, the use of a double (though integrated) system for set-up verification brings about redundancy of control systems for higher safety. In addition, the preliminary correction performed by the OTS is believed to improve the outcomes of the second image-based registration procedure, which is applied for set-up refinement. This last point is essential for session duration minimization, as patient positioning occupies a considerable extent of the duration of a particle therapy session.

One further potential advantage of the use of optical tracking devices as non-ionizing motion detection devices is the ability to check for patient immobility without any extra irradiation for the patient. At CNAO, even very small patient motion can easily be detected during the treatment fraction, given the high frequency of the OTS acquisition, and immediate interruption of the dose delivery can be manually triggered. In addition, OTS systems at CNAO are expected to play an essential role in supporting time-resolved dose delivery strategy in the near future.

## FUNDING

This work was supported by the European Community's Seventh Framework Programme [FP7/2007–2013] under Grant Agreement No. 215840-2.
